# Functional Analyses of a Putative, Membrane-Bound, Peroxisomal Protein Import Mechanism from the Apicomplexan Protozoan *Toxoplasma gondii*

**DOI:** 10.3390/genes9090434

**Published:** 2018-08-29

**Authors:** Alison J. Mbekeani, Will A. Stanley, Vishal C. Kalel, Noa Dahan, Einat Zalckvar, Lilach Sheiner, Wolfgang Schliebs, Ralf Erdmann, Ehmke Pohl, Paul W. Denny

**Affiliations:** 1 Department of Biosciences and Centre for Global Infectious Disease, Stockton Road, Durham DH1 3LE, UK; a.j.mbekeani@durham.ac.uk (A.J.M.); will.stanley2011@gmail.com (W.A.S.); ehmke.pohl@durham.ac.uk (E.P.); 2 Institute of Biochemistry and Pathobiochemistry, Department of Systems Biochemistry, Faculty of Medicine, Ruhr University Bochum, 44780 Bochum, Germany; vishal.kalel@rub.de (V.C.K.); wolfgang.schliebs@rub.de (W.S.); ralf.erdmann@rub.de (R.E.); 3 Department of Molecular Genetics, Weizmann Institute of Science, Rehovot 7610001, Israel; noa.dahan@weizmann.ac.il (N.D.); einat.zalckvar@weizmann.ac.il (E.Z.); 4 Wellcome Centre for Molecular Parasitology, University of Glasgow, Glasgow G12 8TA, UK; lilach.sheiner@glasgow.ac.uk

**Keywords:** Apicomplexa, *Toxoplasma*, peroxisomes, peroxin, Pex5

## Abstract

Peroxisomes are central to eukaryotic metabolism, including the oxidation of fatty acids—which subsequently provide an important source of metabolic energy—and in the biosynthesis of cholesterol and plasmalogens. However, the presence and nature of peroxisomes in the parasitic apicomplexan protozoa remains controversial. A survey of the available genomes revealed that genes encoding peroxisome biogenesis factors, so-called peroxins (Pex), are only present in a subset of these parasites, the coccidia. The basic principle of peroxisomal protein import is evolutionarily conserved, proteins harbouring a peroxisomal-targeting signal 1 (PTS1) interact in the cytosol with the shuttling receptor Pex5 and are then imported into the peroxisome via the membrane-bound protein complex formed by Pex13 and Pex14. Surprisingly, whilst Pex5 is clearly identifiable, Pex13 and, perhaps, Pex14 are apparently absent from the coccidian genomes. To investigate the functionality of the PTS1 import mechanism in these parasites, expression of Pex5 from the model coccidian *Toxoplasma gondii* was shown to rescue the import defect of Pex5-deleted *Saccharomyces cerevisiae*. In support of these data, green fluorescent protein (GFP) bearing the enhanced (e)PTS1 known to efficiently localise to peroxisomes in yeast, localised to peroxisome-like bodies when expressed in *Toxoplasma.* Furthermore, the PTS1-binding domain of Pex5 and a PTS1 ligand from the putatively peroxisome-localised *Toxoplasma* sterol carrier protein (SCP2) were shown to interact in vitro. Taken together, these data demonstrate that the Pex5–PTS1 interaction is functional in the coccidia and indicate that a nonconventional peroxisomal import mechanism may operate in the absence of Pex13 and Pex14.

## 1. Introduction

Peroxisomes were first biochemically characterised and named by Christian de Duve and colleagues following purification from rat liver tissue, and later the protozoa [1,2]. Within the isolated, membrane-bound microbodies, they identified several oxidases that oxidize substrates whilst reducing oxygen to hydrogen peroxide (H_2_O_2_), as well as two enzyme classes able to reduce this damaging H_2_O_2_: peroxidases and catalases [2]. The following decades saw peroxisomes identified as apparently ubiquitous, functionally diverse, eukaryotic organelles involved in many catabolic functions including lipid, amino acid and purine metabolism [3,4]. This biogenesis and functionality of peroxisomes is dependent on a group of proteins known as peroxins (Pex). Furthermore, the topogenic import signals for the import of matrix proteins is conserved across evolution [5]. For most imported proteins this motif comprises a short tripeptide sequence at the extreme C-terminus. Prototypically, this motif, serine–lysine–leucine (-SKL) or neutral variants, is known as peroxisomal-targeting signal 1 (PTS1) [6]. The PTS1 receptor is the peroxin Pex5, a shuttling receptor, which translocates the PTS1-bearing ligand from the cytosol to the peroxisome matrix via interaction with a membrane docking complex of Pex13 and Pex14 (and Pex17 in yeast) [7,8]. 

Illustrative of the essential function of peroxisomes in many eukaryotes is the association of mutations in the Pex5–PTS1 import system with human diseases such as Zellweger syndrome [9]. However, despite the crucial roles these organelles can play, eukaryotic microbes such as the intestinal parasite *Entamoeba histolytica* have been reported to lack peroxisomes, presumably due to their existence in low-oxygen environments and the consequent lack of H_2_O_2_ [10]. Other intracellular parasites such as the fungal Microsporidia and the protozoan Apicomplexa (including the malaria parasite *Plasmodium* spp.), as well as intestinal parasitic helminths, have also been reported to lack peroxisomes and encoded Pex proteins [4,11]. However, as the genome databases have developed it has become clear that some apicomplexan parasites, the coccidia which include *Eimeria* spp. (coccidiosis in poultry) and *Toxoplasma gondii* (toxoplasmosis), encode many of the Pex proteins synonymous with the presence of these organelles [5]. 

Obligate intracellular organisms rely on the host cell for many of their nutritional requirements. For example, *Toxoplasma* is auxotrophic for cholesterol and is reliant on that scavenged from the host via endocytosis of low-density lipoprotein (LDL) [12,13], a process that has been proposed as a possible drug target [14]. This salvage of LDL requires molecular mechanisms for coordinated delivery to the desired organelles, and a sterol carrier protein (SCP2) has been characterised as playing a key role in this process [15]. In the Eukaryota, SCP2 is specifically targeted to peroxisomes via a PTS1, a process important for the β-oxidation of fatty acids [16]. Similarly, the *Toxoplasma* SCP2, which harbours a putative PTS1 (SRL), is localised to peroxisomes in mammalian cells, and to the lumen of well-defined vesicles in the parasite [15]. However, the identity of these vesicles as peroxisomes is controversial, with the predicted PTS1 signal of *Toxoplasma* catalase failing to direct green fluorescent protein (GFP) to membrane-bound microbodies in one study [17]. However, a parallel study showed the parasite catalase localising to vesicles in *Toxoplasma* and to mammalian peroxisomes, with the putative PTS1 signal responsible [18].

Clearly, the nature and/or presence of peroxisomes in the Apicomplexa requires further analyses. However, bioinformatic studies have very recently provided evidence, by virtue of the presence of encoded Pex and PTS-bearing proteins, that *Toxoplasma* and related coccidia harbour peroxisomes [5,19,20]. Here, we have taken a functional biology approach to define the *Toxoplasma* Pex5 as a functional orthologue of the yeast, but not human, protein. In addition, we have shown that the enhanced (e)PTS1 identified from *Saccharomyces cerevisiae* [21] efficiently localises GFP to peroxisome-like bodies when expressed in *Toxoplasma.* Finally, we have biochemically demonstrated that the PTS1 ligand domain of the parasite SCP2 interacts with the PTS1-binding domain from *Toxoplasma* Pex5 in vitro. Taken together with previous studies, these data strongly suggest the presence of peroxisomes in this group of parasites.

## 2. Materials and Methods

### 2.1. *Toxoplasma* Pex5 Complementation of Yeast and Human Mutant Lines

The complete coding region of the *Toxoplasma* protein, *Tg*Pex5 (TGGT1_231870), was synthesised by GenScript (Piscataway, NJ, USA). Using the Infusion system (Takara, Kusatsu, Japan) according to manufacturer’s instructions, this sequence was amplified and cloned into:

(i) the mammalian bicistronic expression vector pIRES2-enhanced(e)GFP-SKL [22] using the primer pair:

F:5′CTCAAGCTTCGAATTCTGATGGCTTTCCGTGCG3′

R:5′GAGGGAGAGGGGCGGATCCGGTTAGACGTTCTTGATC3′

(ii) the yeast expression vector pRS416 (containing the *Sc*PEX5 promoter) [23] using the primer pair:

F:5′CAATATATCATAACACGTCGACTGATGGCTTTCCGTGCG3′ 

R:5′CTTAGCGGCCGCACTAGTAGATCTTTAGACGTTCTTGATCATCCC3′ 

The plasmid resulting from (i), pIRES2-*Tg*Pex5-eGFP-SKL, and pIRES2-*Hs*PEX5-eGFP-SKL as a positive control, were transfected into human skin fibroblast cell line GM5756 T (wild-type and Zellweger patient-derived PEX5-deficient) [24]. Wild-type and mutant cells were maintained in Dulbecco Modified Eagle Media (DMEM; Sigma Aldrich, St. Louis, MO, USA) supplemented with 10% Fetal Bovine Serum (FBS; ThermoFisher Scientific, Waltham, MA, USA), 4 mM L-glutamine (Sigma Aldrich), 1% penicillin/streptomycin (Sigma Aldrich) at 37 °C, 5% CO_2_. Transfection was carried out using the X-tremeGENE 9 DNA transfection kit (Roche, Basel, Switzerland) according to the manufacturer’s protocol and the cells incubated for 72 h. 

The plasmid resulting from (ii), pRS416-*Tg*Pex5, and pRS416-*Sc*Pex5 as a positive control (both containing *Sc*PEX5 promoter), were transformed into *Saccharomyces cerevisiae* UTL-7A (wild-type and *pex5* deletion strains) for growth assay, or co-transformed with pRS415-based plasmid encoding GFP-SKL as a PTS1 peroxisomal marker for microscopy [23]. For transformation, yeast were grown to exponential phase and transformed as previously described [25,26,27,28,29,30]. Single or double transformants were selected on −Ura or −Ura−Leu plates, respectively, and later cultured in selective liquid media containing 0.3% (*w*/*v*) glucose or 0.1% (*w*/*v*) oleic acid as previously described [31].

After incubation, human cells were washed with Dulbecco Phosphate-Buffered Saline (DPBS; Sigma Aldrich) and fixed in 4% *v*/*v* paraformaldehyde (Roth, Karsruhe, Germany) in DPBS for 20 min at room temperature. Cells fixed on cover-slips were then mounted on glass slides under the anti-fade reagent Mowiol (Sigma Aldrich) containing 4′,6-ciamidino-2-phenylindole (DAPI; ThermoFisher Scientific). Yeast cells were directly visualized under microscopy. For imaging, wide-field fluorescence microscopy was performed using an Axioplan 2 microscope with an AxioCam MR digital camera and Axiovision software version 4.6.3 (Zeiss, Oberkochen, Germany). Green fluorescent protein signal was visualized using 450–490 nm band pass excitation filter, a 510 nm dichromatic mirror and a 515–565 nm band pass emission filter.

### 2.2. Growth Analyses of *Toxoplasma* Pex5 Complementation of Yeast Mutant Line

Serial 10-fold dilutions of exponentially growing UTL-7A *Pex5* deleted yeast, transformed with *Tg*Pex5, *Sc*Pex5 or empty vector, were spotted onto −Ura glucose or oleate plates and incubated at 30 °C for 3 to 4 days before image capture as previously described [31,32].

### 2.3. Localisation of Enhanced PTS1-Tagged GFP in *Toxoplasma*

The GFP-enhanced(e)PTS1 cassette was amplified from the vector pFA6a-GFP-eSKL [21] using the primer pair:

F:5′GAAAACTACTCGTTGGCATTTTTTCTTGAATTCCATGAGTAAAGGAGAAGAACTTTTCACTGG3′

R:5′GAGAAGTGAGCACAACGGTGATTAATTAACTACAATTTGGATCTTCTACCTCTTCCCAATG3′

Using the restriction-free methodology [33], following purification, the PCR product was cloned into pTUB8-myc-GFP to form pTUB8-GFP-ePTS1.

*Toxoplasma* RH strain were transfected with pTUB8-GFP-ePTS1 and used to infect human foreskin fibroblasts cultured on glass coverslips as previously described [29]. After 24 h, cells were fixed with 4% paraformaldehyde (20 min, room temperature) and washed in PBS, before permeabilization and blocking in PBS/0.02% Triton-X-100/2% Bovine Serum Albumin (BSA; 20 min, room temperature). Cells were then incubated for 60 min at room temperature with the primary antibodies (anti-IMC1 [34] and anti-GFP, Roche) diluted 1:1000, washed three times, and incubated with Alexa488 goat anti-mouse and Alexa594 goat anti-rabbit (ThermoFisher Scientific; 45 min, room temperature), all in PBS/0.02% Triton-X-100/2% BSA. 

Images were obtained using a DeltaVision Core microscope (GE Heathcare, Chicago, IL, USA) and processed using Softworx (GE Heathcare) and FIJI software [35]. Parasites with heavily distorted cell shape and fragmented mitochondria were excluded from the analysis.

### 2.4. Analyses of the Interaction of *Toxoplasma* Pex5 and SCP2

Regions encoding domains for the predicted PTS1 of *Tg*SCP2 (*Tg*SCP2_PTS1_: *Tg*SCP2 499-625) and the PTS1-binding region of receptor *Tg*Pex5 (*Tg*Pex5C: *Tg*Pex5 581-899) were synthesized with an *Escherichia coli* codon bias and cloned into the bacterial expression vector pETM-30 by GenScript. The resulting pETM-30-*Tg*SCP2_PTS1_ and pETM-30-*Tg*Pex5C were transformed into *E. coli* BL21 (DE3) (ThermoFisher Scientific), and grown in pH 7.5 buffered Luria-Bertani (LB) medium, supplemented with 1% (*w*/*v*) glucose and 50 mg/mL kanamycin (Sigma-Aldrich) at 37 °C. When exponential growth was reached, the temperature was reduced to 20 °C for 1 h, before induction with 0.5 mM IsoPropyl β-D-1-ThioGalactopyranoside (IPTG; Sigma Aldrich) and overnight incubation at 30 °C. Cell pellets were flash frozen before resuspension in Buffer A: 100 mM KH_2_PO_4_ pH 7.4, 5 mM 2-mercaptoethanol, EDTA-free complete protease inhibitor cocktail, 1 mg/mL lysozyme, 1 mg/mL DNase I and 1 mg/mL RNAse A (Sigma-Aldrich). The bacteria were then sonicated (Electronic UW2200; Bandelin, Berlin, Germany) and, following centrifugation, the supernatant collected through a 0.45 µm filter (Sigma Aldrich). This supernatant was then applied to a 1 mL Glutathione Sepharose 4B resin column (GSTrap) (GE Healthcare), which was pre- equilibrated with Buffer A. Following a Buffer A wash, the protein was eluted with Buffer A containing 20 mM reduced glutathione. The *Tg*Pex5C HIS-GST-tag was cleaved using TEV protease (Sigma Aldrich) in accordance with manufacturer’s instructions, before dialysis into Buffer A and application to a 1 mL Ni-NTA (ThermoFisher Scientific) column equilibrated in Buffer A and eluted with Buffer A supplemented with 250 mM imidazole, pH 7.4. To analyse the interaction, *Tg*SCP2 PTS1 containing His_6_(HIS)-GST-tag was bound to a GSTrap column and isolated *Tg*Pex5C applied in Buffer A. Following a Buffer A wash, the proteins were eluted with Buffer A containing 20 mM reduced glutathione before analyses by SDS–PAGE. 

## 3. Results

### 3.1. Functional Complementation Analyses of *Tg*Pex5 in Yeast and Human Cell Lines

**Figure 1:**
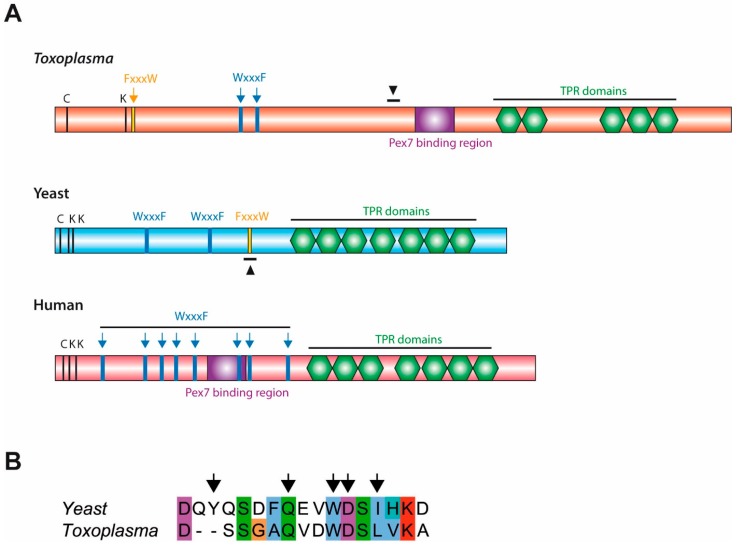
(**A**) *Toxoplasma* Peroxin 5 (Pex5) contains structural characteristics conserved in yeast and human Pex5. Bioinformatic comparison of the *Toxoplasma* Pex5 protein sequence with yeast and human counterparts indicated the presence of critical cysteine and lysine residues (C, K), pentapeptide motifs (WxxxF/FxxxW) and structural domains (Pex7-binding region and TetratricoPeptide Repeat [TPR] domains which bind peroxisomal-targeting signal 1 [PTS1] ligands) characteristic of Pex5. Additionally, a region homologous to the Fox1 binding site in yeast Pex5 is also found in *Toxoplasma* Pex5; indicated by black arrowheads. (**B**) Sequence alignment of the Fox1 binding site in yeast Pex5 (*Sc*Pex5_251-267_) with *Toxoplasma* Pex5 (*Tg*Pex5_442-456_), arrows indicate positions in *Sc*Pex5 critical for Fox1 binding [36].

**Figure 2:**
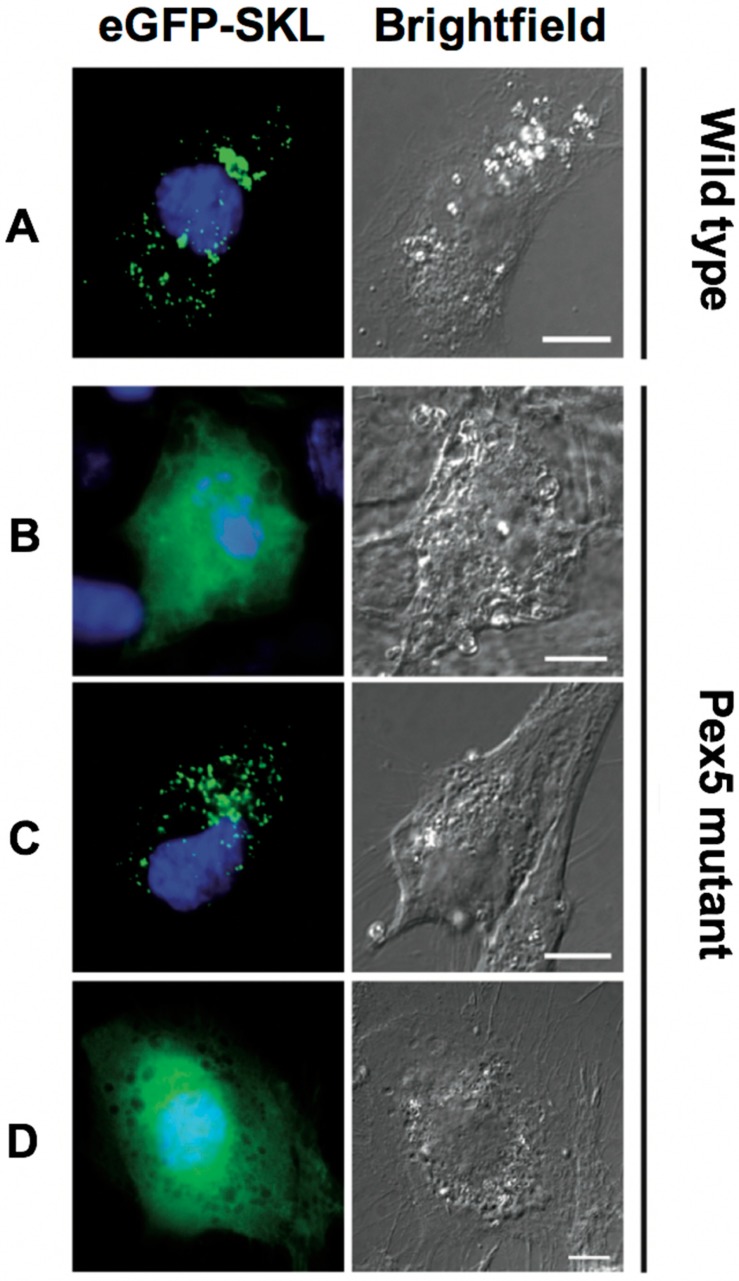
*Tg*Pex5 does not functionally replace Pex5 function in human fibroblast cells. In wild-type cells (**A**), peroxisomal reporter enhanced (e) GFP-SKL is imported into peroxisomes, resulting in a characteristic punctuate pattern. In Pex5 mutant cells (**B**, negative control), eGFP-SKL is mislocalised to the cytosol, resulting in the diffuse cytosolic pattern of GFP. Co-expression of human Pex5 (*Hs*Pex5L, **C**, positive control) restored the localisation of eGFP-SKL to peroxisomes. In contrast, co-expression of *Tg*Pex5 (**D**) did not localise eGFP-SKL to peroxisomes. Scale bar is 10 µm.

**Figure 3:**
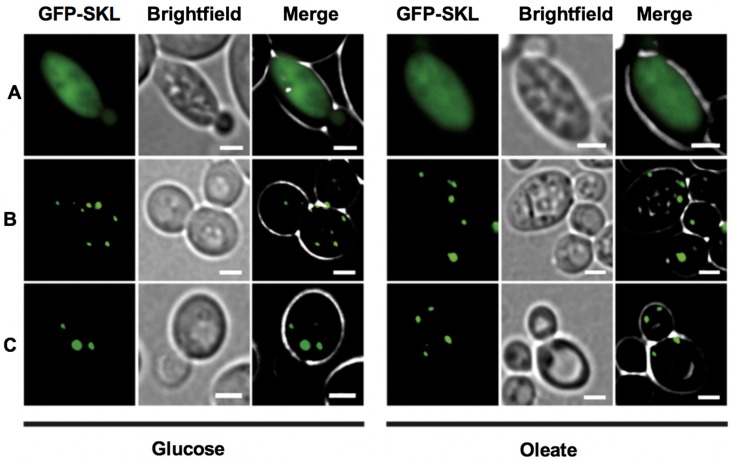
*Tg*Pex5 complements for the loss of Pex5 in Δ*pex5* mutant yeast when grown on medium containing either glucose or oleate as the sole carbon source. No complementation is indicated by a mislocalization of the heterologously expressed GFP-SKL to the cytosol as seen for the empty vector control (**A**). Functional complementation restored peroxisomal import of the GFP-SKL and led to the appearance of a punctate pattern, as seen upon expression of the gene coding for yeast Pex5 (**B**), and the gene encoding*Tg*Pex5 (**C**). These results indicated that *Tg*Pex5 can functionally replace the yeast Pex5p in PTS1-protein import into peroxisomes. Scale bar is 5 µm.

**Figure 4:**
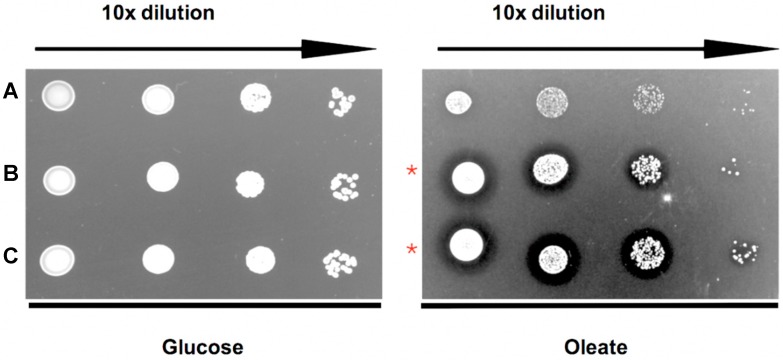
*Tg*Pex5 expression complements the growth defect of yeast Δ*pex5* mutant cells on oleic acid media. (**A**) Empty vector negative control; (**B**) *Sc*Pex5 positive control; and (**C**) *Tg*Pex5. 10-fold dilutions (from the left) spotted onto either glucose and oleate plates. As expected, all strains grow well in the presence of glucose. On oleate medium, the growth of the Δ*pex5* mutant transformed with the empty vector control is affected. In contrast, Δ*pex5* mutant cells transformed with plasmids coding for either *Sc*Pex5 (**B**) or *Tg*Pex5 (**C**) showed enhanced growth and a halo that is characteristic of efficient oleate utilisation and peroxisomal oxidation (red stars).

Bioinformatic analyses of *Toxoplasma*, yeast and human Pex5 protein sequences indicated that parasite Pex5 contains structural features characteristic of Pex5 proteins. These include (i) C-terminal TetratricoPeptide Repeat (TPR) motifs forming the PTS1-binding site; (ii) within the probably unstructured N-terminal domain, three so-called WxxxF motifs required for receptor docking; and (iii) a single cysteine at a conserved position, which becomes ubiquitinated to initiate recycling of the receptor into the cytosol (Figure 1A). *Toxoplasma* Pex5 was heterologously expressed in human or yeast cells for functional complementation analysis. In contrast to the positive control, human *Hs*Pex5L [24], expression of *Toxoplasma Tg*Pex5 in Pex5-deficient human fibroblasts was unable to restore the PTS1-mediated trafficking of eGFP to peroxisomal compartments (Figure 2). However, parallel analyses in *S. cerevisiae* demonstrated that *Tg*Pex5 could clearly restore GFP-PTS1 targeting to peroxisomes in a cellular context (Figure 3). The results of this localisation assay were augmented by the observation that *Tg*Pex5, like *Sc*Pex5 [23], was able to restore peroxisomal function and β-oxidation as characterised by increased growth and the utilisation of fatty acid oleate, which results in halo formation around the colonies on oleate plates (Figure 4) [31,32]. Growth complementation indicates that the heterologous *Tg*Pex5 targets not only the artificial cargo GFP-PTS1, but also the endogenous yeast enzymes, into peroxisomes. It is remarkable that *Tg*Pex5 restored translocation into the organellar lumen of not only the β-oxidation enzymes with typical PTS1, but also of the non-PTS acyl-CoA oxidase Fox1, the initial enzyme required for the fatty acid catabolic pathway. The Fox1 binding regions have been assigned to the N-terminal half of *Sc*Pex5 [36]. Sequence alignments between yeast and *Toxoplasma* Pex5 suggest that the key residues required for Fox1 interaction are largely conserved (Figure 1B). Taken together, these data indicate that *Toxoplasma Tg*Pex5 is a functional orthologue of the yeast Pex5, and heterologous expression of this protein can functionally replace its yeast but not its human counterpart. 

### 3.2. Localization of GFP Tagged with Enhanced PTS1 (GFP-ePTS1) in Toxoplasma

**Figure 5:**
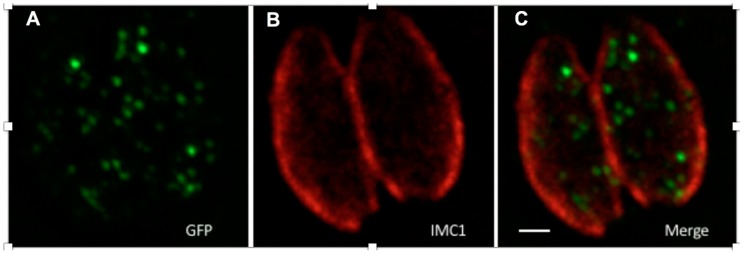
GFP tagged with ePTS1 localised in peroxisome-like bodies when transiently expressed in *Toxoplasma.* The image shows two parasites within a human fibroblast vacuole, GFP-ePTS1 stained with anti-GFP antibody (**A**, green), the parasite pellicle stained with anti-IMC1 antibody (**B**, red), and a merge of A and B (**C**). The scale bar is 1 µM.

Enhanced (e)PTS1 was identified by screening a randomised library for linker sequences (directly upstream of PTS1) for the ability to more efficiently localise fluorescent protein to peroxisomes in *S. cerevisiae* [21]. When GFP-ePTS1 was expressed in *Toxoplasma*, it localised to punctate structures with diameters ranging from 129–311 nm (Figure 5), this concurs with the diameter of peroxisomes (100–200 nm) and previous observations in this parasite [15,18]. This observation supports (i) the interaction of PTS1 (from yeast, as well as *Toxoplasma*) with Pex5 in the parasite; (ii) the existence of peroxisomes with the canonical translocation machinery in the coccidia.

### 3.3. In Vitro Analyses of the Interaction of *Tg*Pex5 and *Tg*SCP2

**Figure 6:**
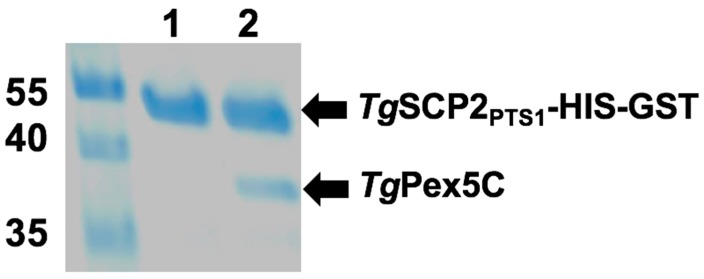
Interaction of *Tg*SCP2 PTS1 and *Tg*PEX5C in vitro. Pull-down assay was performed using the *bait Tg*SCP2 PTS1 with a HIS-GST tag. Eluates of the pull-down without prey protein (Lane 1, negative control) and *Tg*PEX5C (Lane 2) are shown. Molecular weight markers are in kilodaltons.

Regions encoding the following domains were expressed in *E. coli*: (i) predicted *Tg*SCP2 PTS1 (*Tg*SCP2_PTS1_: *Tg*SCP2 499–625) containing the PTS1-targeting signal; and (ii) *Tg*Pex5 PTS1 receptor (*Tg*Pex5C: *Tg*Pex5 581–899), harbouring the predicted PTS1-binding domain. The recombinant proteins were isolated and subjected to in vitro binding studies. Using HIS-GST-tagged *Tg*SCP2 PTS1 (43.4 kDa) bound to a GSTrap column as *bait*, the binding of *Tg*Pex5C (34.5 kDa) was analysed. Following stringent washes, the elution of bound material demonstrated interaction with *Tg*SCP2_PTS1_, showing that the *Toxoplasma* Pex5 can bind to the parasite PTS1 (Figure 6).

## 4. Discussion

With recent bioinformatic analyses demonstrating the presence of a suite of Pex proteins in the coccidian apicomplexans, but not in other subclasses [5,19,20], functional analyses are demanded. However, previous studies in *Toxoplasma* have proven to be controvertible. The *Toxoplasma* catalase was localised to peroxisome-like vesicles in one study [18], however, in the same year it was reported that the putative catalase PTS1 failed to direct GFP to these membrane-bound microbodies [17]. The sterol carrier protein SCP2 has also been shown to localise to peroxisome-like bodies in *Toxoplasma* [15].

To improve the molecular understanding of the putative peroxisomal bodies in *Toxoplasma* and the related coccidians, here we report a series of cellular and biochemical studies of the parasite Pex5 orthologue. Like human, plant and nematode [37], the *Toxoplasma* protein clearly rescues the targeting of GFP bearing a canonical PTS1 sequence (SKL) in yeast, indicating that *Tg*Pex5 is a functional peroxin involved in the trafficking of PTS1-bearing proteins to the peroxisomes. In addition, yeast-derived ePTS1 was sufficient to localise GFP to peroxisome-like bodies in *Toxoplasma* [15,18], and *Tg*SCP_PTS1_ (-SRL) clearly binds to the parasite *Tg*Pex5 PTS1-binding domain in vitro. Furthermore, heterologous expression of *Tg*Pex5 restored the utilisation of oleate in Pex5-deficient yeast, clearly demonstrating that a complete set of endogenous fatty acid-degrading enzymes with, and without, typical PTS1 sequences are recognized and targeted to peroxisomes by the parasite peroxin. This common cargo selectivity of the yeast and *Toxoplasma* receptor will include Fox1, which is recognized by a PTS1-independent binding domain N-terminal of the yeast Pex5 PTS1-binding region. Importantly, unlike the mammalian orthologue, *Tg*Pex5 maintains a Fox1-binding region. In contrast to yeast, *Tg*Pex5 was unable to direct eGFP-SKL to human fibroblast peroxisomes and thus rescue the import defect of an equivalent mammalian Pex5-deficient cell line. Taken together, these data indicate that *Tg*Pex5 is a functional orthologue of the yeast but not the human peroxin. A difference that may be exploited by consideration of the *Tg*Pex5-PTS1 interaction as a drug target, as has been described in the protozoan pathogen *Trypansoma brucei* [38]. 

Therefore, in summary we have demonstrated that the *Toxoplasma* Pex5 is functional in the context of a ∆*pex5* yeast strain and interacts with the protozoan PTS1 in vitro. Coupled with the localization of ePTS1-tagged GFP to peroxisome-like bodies in *Toxoplasma,* this strongly suggests that the coccidia harbour peroxisomes. The relative lack of structural evidence may be due to lifecycle-dependent expression, with the peroxins characterised by upregulation during the extracellular stages of the lifecycle, oocysts and sporozoites. Future studies should be directed here, rather than at the more tractable, intracellular, tachyzoite forms [20]. In addition, in all eukaryotic systems studied to date, a membrane-bound docking complex of Pex13 and Pex14 is required for peroxisomal protein import [7,8]. However, an obvious Pex13 is missing in the genome of *Toxoplasma* and related coccidian parasites [19,20]. Furthermore, the identity and presence of Pex14 in *Toxoplasma* is disputed [5,19,20]. Therefore, the exact mode of Pex5-mediated protein import into peroxisomes in the coccidia is unclear and further bioinformatic and functional analyses are warranted.
